# HIV-1 proteins gp120 and tat induce the epithelial–mesenchymal transition in oral and genital mucosal epithelial cells

**DOI:** 10.1371/journal.pone.0226343

**Published:** 2019-12-23

**Authors:** Kathy Lien, Wasima Mayer, Rossana Herrera, Kristina Rosbe, Sharof M. Tugizov

**Affiliations:** 1 Department of Medicine, University of California–San Francisco, San Francisco, CA, United States of America; 2 Department of Otolaryngology, University of California–San Francisco, San Francisco, CA, United States of America; 3 Department of Obstetrics, Gynecology & Reproductive Sciences, University of California–San Francisco, San Francisco, CA, United States of America; Emory University, UNITED STATES

## Abstract

The oral, cervical, and genital mucosa, covered by stratified squamous epithelia with polarized organization and strong tight and adherens junctions, play a critical role in preventing transmission of viral pathogens, including human immunodeficiency virus (HIV). HIV-1 interaction with mucosal epithelial cells may depolarize epithelia and disrupt their tight and adherens junctions; however, the molecular mechanism of HIV-induced epithelial disruption has not been completely understood. We showed that prolonged interaction of cell-free HIV-1 virions, and viral envelope and transactivator proteins gp120 and tat, respectively, with tonsil, cervical, and foreskin epithelial cells induces an epithelial–mesenchymal transition (EMT). EMT is an epigenetic process leading to the disruption of mucosal epithelia and allowing the paracellular spread of viral and other pathogens. Interaction of cell-free virions and gp120 and tat proteins with epithelial cells substantially reduced E-cadherin expression and activated vimentin and N-cadherin expression, which are well-known mesenchymal markers. HIV gp120- and tat-induced EMT was mediated by SMAD2 phosphorylation and activation of transcription factors Slug, Snail, Twist1 and ZEB1. Activation of TGF-β and MAPK signaling by gp120, tat, and cell-free HIV virions revealed the critical roles of these signaling pathways in EMT induction. gp120- and tat-induced EMT cells were highly migratory via collagen-coated membranes, which is one of the main features of mesenchymal cells. Inhibitors of TGF-β1 and MAPK signaling reduced HIV-induced EMT, suggesting that inactivation of these signaling pathways may restore the normal barrier function of mucosal epithelia.

## Introduction

The oropharyngeal, ectocervical, vaginal, and foreskin epithelia consist of a multilayered, stratified squamous epithelium supported by an underlying layer of fibrous connective tissue, the lamina propria. The endocervical and intestinal mucosa are covered with monostratified simple epithelium. All mucosal epithelia form multiple intercellular junctions, including tight and adherens junctions [1–10], which are critical for maintaining the morphologic and physiologic features of mucosal epithelia, including their barrier functions. Tight junctions of mucosal epithelium form the physical tissue barrier between epithelial cells that protects the internal body from the penetration of external infectious agents [11], including pathogenic viruses.

In individuals with HIV-caused acquired immunodeficiency syndrome (AIDS), tight junctions in oral, intestinal, and genital mucosal epithelia are disrupted, leading to impairment of mucosal functions [7, 12–18]. In vitro studies show that the interaction of HIV proteins gp120 and tat with mucosal epithelia may disrupt tight and adherens junctions of epithelial cells, reducing their barrier functions [7, 19–26].

We have shown that prolonged interaction of HIV envelope protein gp120 and transactivator protein tat with oral and genital epithelia reduces the expression of tight junction proteins occludin and zonula occludens-1, claudin-1, and adherens junction protein E-cadherin, leading to depolarization of epithelial cells [7, 19, 21, 22]. Downregulation of proteins of adherence and tight junctions of epithelial cells and their depolarization may lead to an epithelial–mesenchymal transition (EMT) [27–29].

EMT is a normal multistep epigenetic process in embryonic development that regulates the differentiation of cell lineage identity [30–32]. However, the EMT phenotype also plays an important role in neoplastic processes, facilitating growth, migration and metastasis of tumor cells [30, 33–39]. During cancer-associated EMT, epithelial cells lose cell-cell junctions and become proliferative and invasive [40]. The TGF-β signaling pathway is the dominant canonical regulatory network for this process [41, 42]. Binding of mature TGF-β to TGF-β1 R2 activates TGF-β signaling, leading to activation of downstream molecules, including Smad family transcription factor complexes [43]. These complexes activate the transcriptional regulators Snail, Slug, and Twist1. Activation of Snail and Twist1 may lead to activation of other transcription factors, ZEB1 and ZEB2 [44]. Cooperation between these transcription factors leads to downregulation of E-cadherin and cytokeratin and upregulation of vimentin, fibronectin, and N-cadherin expression [45–49]. Expression of fibronectin is critical for invasion of cancer cells [50–52]. N-cadherin expression plays an important role in the transmigration of cancer cells via endothelial cells, promoting spread and metastasis of neoplastic cells via blood circulation [53–55]. Overexpression of Snail also represses expression of tight junction proteins claudins and occludin-1, leading to depolarization of epithelial cells and EMT [27]. TGF-β may activate Ras-MAPK signaling pathways, which also play a critical role in EMT induction by phosphorylation of Smad2/3 and TWIST1 [56–63]. Crosstalk between TGF-β and MAPK signaling is highly critical for induction and maintenance of the EMT phenotype [64].

The incidence of HPV-associated oropharyngeal cancer is increased in HIV-infected individuals [65–74]. HIV-positive individuals have about a sixfold greater risk for oropharyngeal and tonsillar cancers [75–79] than do uninfected individuals. In addition to oral cancer, the incidence of HPV-associated anal and cervical cancer is 80 and 22 times higher, respectively, in HIV-infected individuals than in uninfected individuals [80–84]. Thus, in HIV- and HPV-coinfected individuals, HIV-induced EMT may accelerate the HPV neoplastic process by increasing the paracellular spread of HPV and the invasion of HPV-infected malignant cells.

The primary goal of this study was to investigate the role of HIV proteins gp120 and tat in the induction of EMT in tonsil, cervical, and foreskin epithelial cells. We show here that exposure of normal tonsil, cervical, and foreskin keratinocytes to tat and gp120 and to cell-free virions for several days leads to the development of the EMT phenotype in these cells, including activation of TGF-β1 and vimentin expression and reduction of E-cadherin expression. HIV-induced EMT of oral and genital epithelium may play a critical role in reducing the barrier function of these epithelia, which may allow the penetration of various viral, bacterial, and fungal pathogens through oral and genital mucosal epithelia.

## Results

### HIV-associated EMT in oral mucosal epithelial cells of HIV/AIDS patents

**Fig 1:**
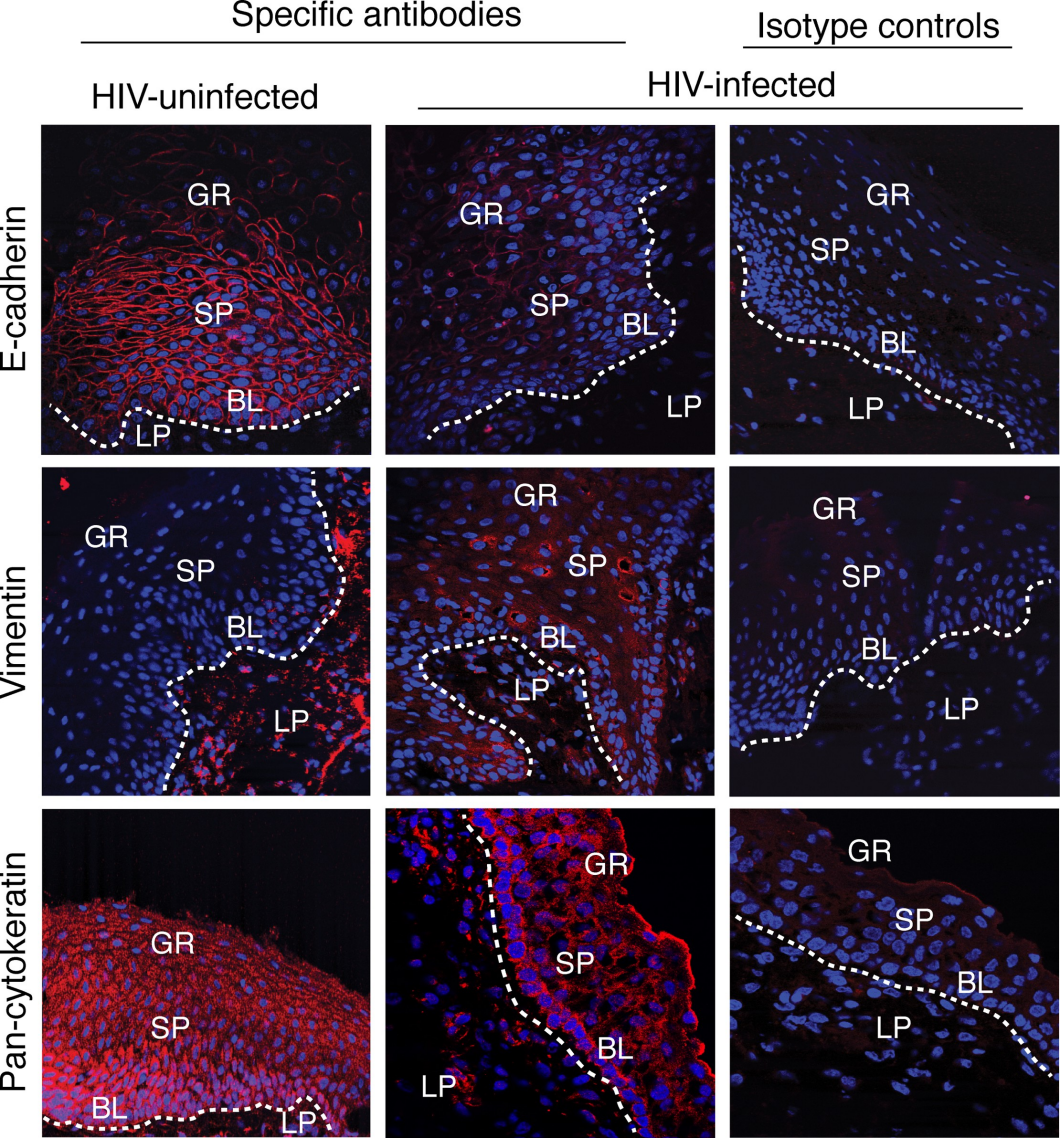
Oral epithelium of HIV-positive individuals has the EMT phenotype. Buccal tissues from HIV-infected and uninfected individuals were immunostained for E-cadherin, vimentin, and pancytokeratin expression (red). Nuclei are stained in blue. GR, granulosum; SP, spinosum; BL, basal; LP, lamina propria. Original magnification: x400. Representative immunofluorescence images are shown.

**Fig 2:**
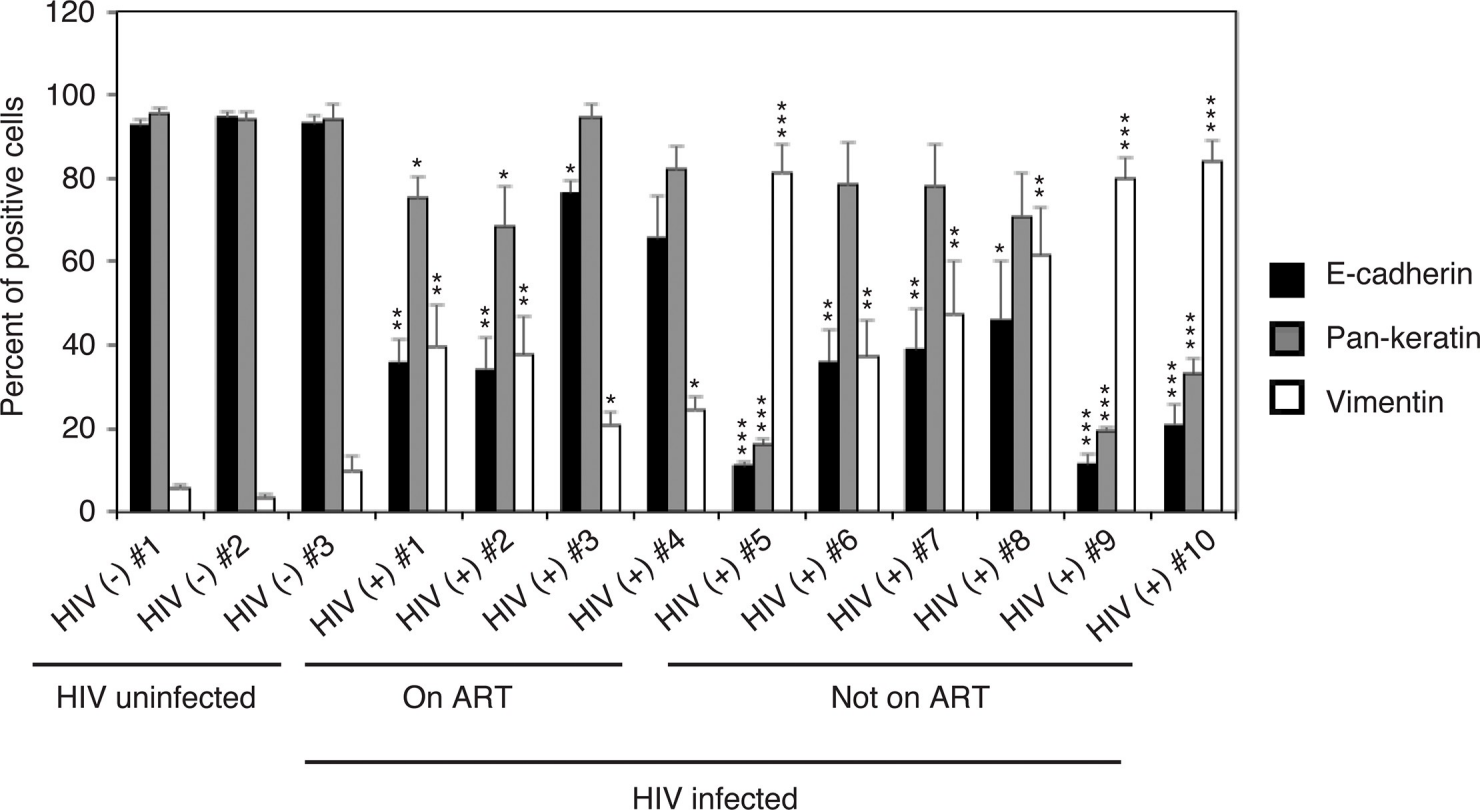
Quantitative analysis of EMT markers in oral epithelium of HIV-infected and uninfected individuals. For quantitative evaluation of EMT marker expression in oral tissues of HIV-infected and uninfected individuals, epithelial cells expressing E-cadherin, vimentin, and pancytokeratin were counted from 10 randomly selected regions of mucosal epithelia. Results are presented as a percentage of epithelial cells expressing E-cadherin, vimentin, and pancytokeratin. Data are shown as the mean ± SD (n = 10). *P<0.05, **P<0.01, ***P<0.001. E-cadherin, pancytokeratin and vimentin expression were compared in ART-treated or untreated samples from HIV-infected individuals with HIV uninfected (control) samples.

We have shown that the oral epithelia of HIV-infected individuals have disrupted tight junctions [7] and that HIV tat/gp120 induces disruption of tight and adherens junctions of keratinocytes in vitro [7, 21]. Since disruption of epithelial junctions is one of the critical features of EMT [31, 35, 85], we immunostained buccal tissues from 10 HIV-infected and 3 uninfected individuals for E-cadherin, pancytokeratin, and vimentin. Four HIV-infected individuals were receiving antiretroviral treatment (ART) (#1- #4), and 6 were not receiving ART treatment (#5 - #10) (Figs 1 and 2).

Three buccal epithelia of uninfected donors showed expression of E-cadherin and pancytokeratin, but not vimentin, in all epithelial cells (Figs 1 and 2), which is characteristic of normal mucosal epithelia. E-cadherin expression in buccal epithelia of HIV-infected individuals receiving ART was reduced by 20–50% compared to uninfected epithelia. A significant reduction of pancytokeratin expression was not detected. However, these tissues showed induction of vimentin expression in 10–40% of epithelial cells (Fig 2). Buccal tissues from HIV-infected individuals not receiving ART also showed a reduction of E-cadherin and pancytokeratin expression. A substantial reduction of pancytokeratin expression was detected in 3 of 6 tissues (50%) from HIV-infected individuals without ART. In these 3 tissues (#5, #9 and #10), 60–80% of cells lost E-cadherin and pancytokeratin expression. This was well correlated with a substantial induction of vimentin expression conforming to EMT phenotype in these epithelia. Moreover, EMT induction of all 3 tissues was correlated with higher viral load (S1 Table) and in 2 of them (#9 and #10) also was correlated with low CD4 count.

**Fig 3:**
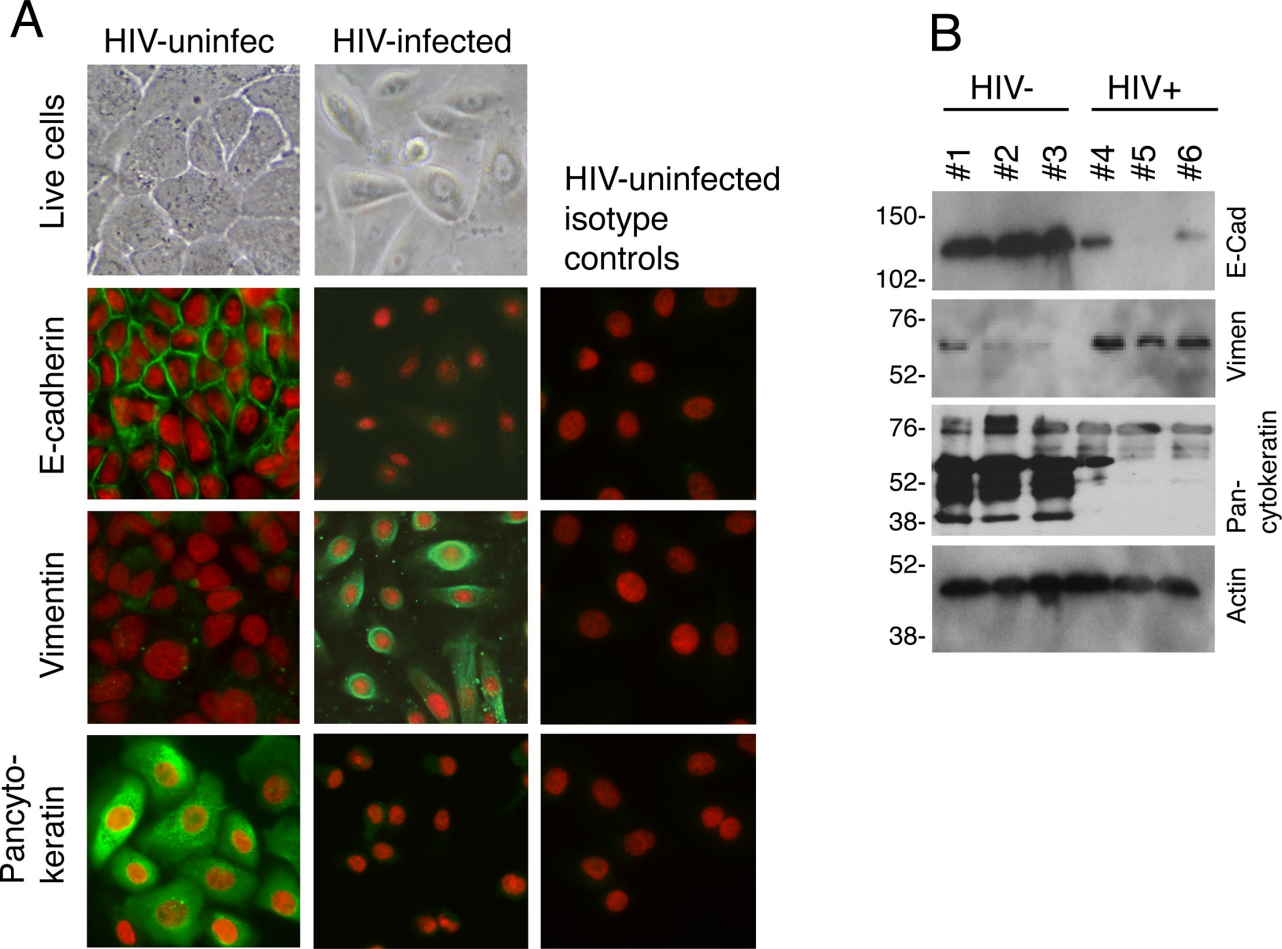
Oral keratinocytes isolated from HIV-infected individuals have an EMT phenotype. (A) Buccal keratinocytes propagated from HIV-infected and uninfected individuals were subjected to phase-contrast microscopy (upper panel) and immunostained for E-cadherin, vimentin, and pancytokeratin (lower panels). (B) Oral keratinocytes from 3 HIV-negative and 3 HIV-infected individuals were examined for E-cadherin, vimentin, and pancytokeratin by Western blot assay.

To confirm the HIV-induced EMT phenotype of oral mucosal epithelial cells, we isolated buccal keratinocytes from 3 uninfected and 3 HIV-infected individuals not receiving ART. Phase-contrast microscopy of oral keratinocytes showed that cells from uninfected individuals had cobblestone-like morphology of epithelial cell sheets with tightly connected cell borders. In contrast, keratinocytes from HIV-infected individuals had a spindle-like shape with weakly connected cell borders, which is typical of the EMT phenotype (Fig 3A).

Immunostaining of keratinocytes for E-cadherin and vimentin showed that oral keratinocytes from uninfected individuals had E-cadherin expression but no vimentin expression (Fig 3A). In contrast, oral keratinocytes from HIV-infected individuals showed a loss of E-cadherin and pancytokeratin and upregulation of vimentin (Fig 3A). These results were confirmed by Western blot assay (Fig 3B).

### HIV proteins tat and gp120 induce the EMT phenotype in oral and genital epithelial cells

To determine the role of HIV-1 in EMT induction, we cultured normal tonsil keratinocytes from uninfected individuals with HIV tat or gp120 proteins at their physiologically relevant concentrations (10 ng/ml each) [86–89], as described in our previous work [7, 21]. In parallel experiments, cells were treated with biologically inactive mutant tat lacking its basic arginine-rich domain and RGD motif [90–92]. HIV gp120 was heat-inactivated. Keratinocytes were maintained for 5 days with viral proteins, and culture medium was changed daily to add fresh virus or proteins.

**Fig 4:**
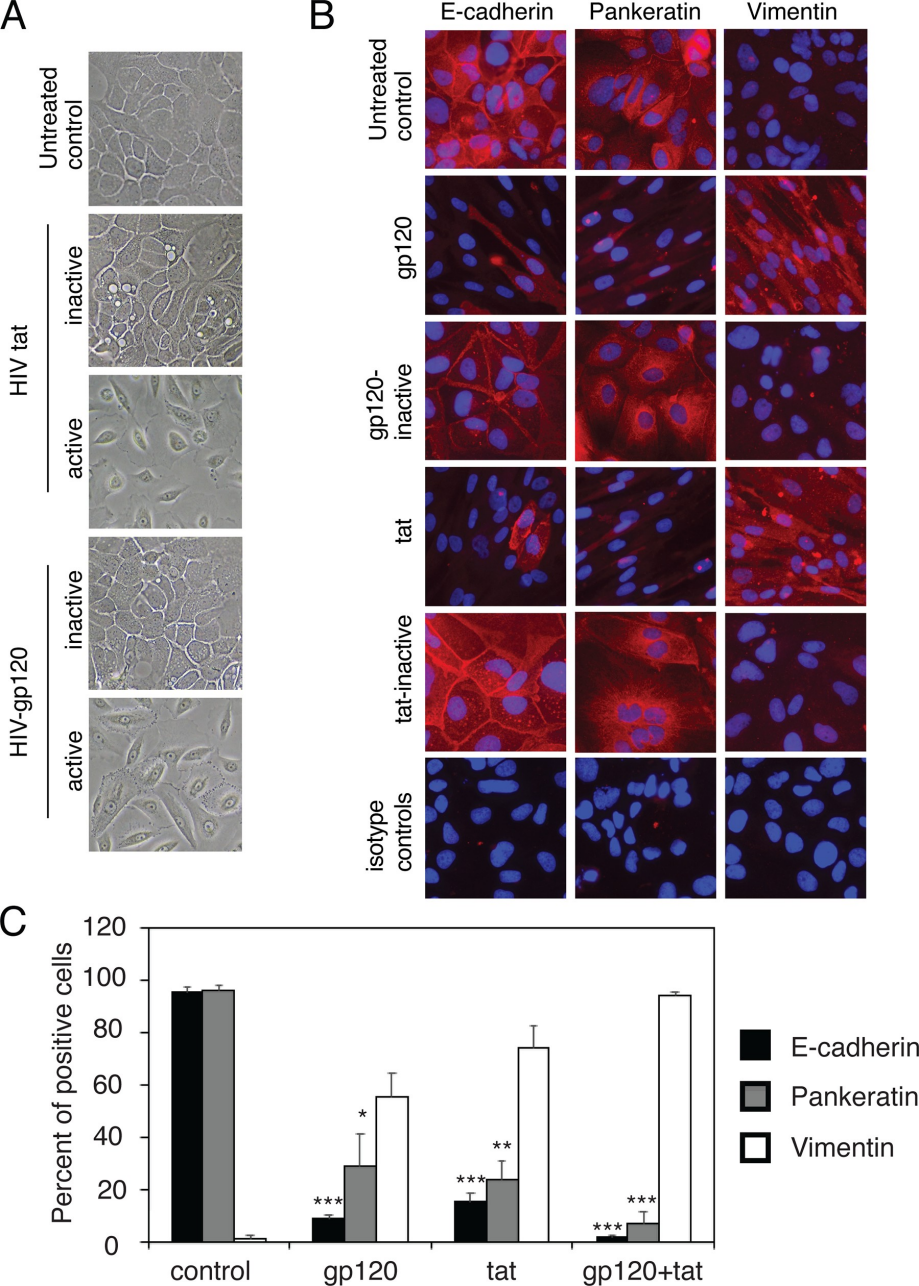
HIV proteins tat and gp120 induce the EMT phenotype in normal oral keratinocytes isolated from uninfected individuals. (A) Normal tonsil keratinocytes isolated from HIV-negative donors were untreated or treated with HIV tat and gp120; their inactive forms were tested independently. After 5 days the live cells were examined using phase-contrast microscopy. (B) Tonsil keratinocytes treated as described in panel A were immunostained for E-cadherin, vimentin, and pancytokeratin. (C) Tonsil keratinocytes untreated or treated with HIV-1 gp120 and/or tat, and cells expressing E-cadherin, vimentin, and pancytokeratin were counted and presented as a percentage of cells expressing E-cadherin, vimentin, and pancytokeratin. Data are representative of 3 independent experiments using tonsil epithelial cells derived from three donors and are shown as the mean ± SD (n = 10). *P<0.05, **P<0.01, ***P<0.001. E-cadherin and pancytokeratin expression were compared with the gp120- and tat-treated and untreated (control) groups.

This treatment should reflect the in vivo prolonged HIV interaction with cells/tissues, because in HIV/AIDS disease, virus progeny are detected in the blood/plasma, saliva, and cervicovaginal secretions; i.e., virions may interact with cells/tissues for days, months, or years. Phase-contrast microscopy of tonsil epithelial cells showed that untreated cells and cells treated with control tat and gp120 had normal epithelial morphology (Fig 4A). In contrast, tonsil keratinocytes treated with active tat and gp120 showed extensive changes in cell morphology, with a spindle-like shape.

To examine the expression of epithelial and mesenchymal markers, at day 5 after treatment we immunostained tonsil cells for E-cadherin, pancytokeratin, and vimentin. Immunofluorescence microscopy showed that untreated tonsil cells or tonsil cells treated with inactive tat and gp120 showed expression of E-cadherin and pancytokeratin, but not vimentin (Fig 4B). However, cells treated with active tat and gp120 inhibited E-cadherin and pancytokeratin expression but upregulated vimentin expression (Fig 4B). Quantitative analysis of E-cadherin and pancytokeratin expression showed that treatment of cells with tat and gp120 led to reduction of E-cadherin and pancytokeratin expression in 70–90% of treated cells compared to control cells (Fig 4C). Induction of vimentin expression in cells treated with active tat and gp120 reached 60–70%. Inhibition of E-cadherin and pancytokeratin and induction of vimentin expression by a combination of tat and gp120 reached ~90%.

**Fig 5:**
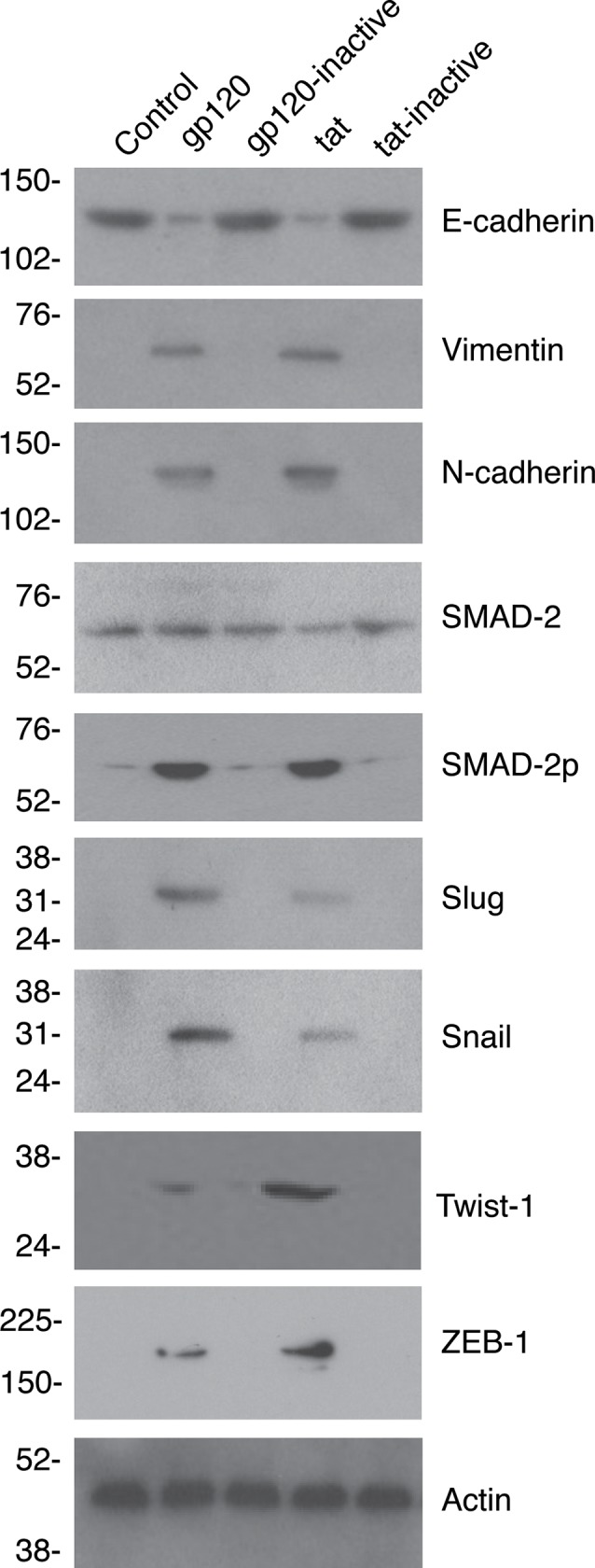
HIV gp120 and tat induce activation of EMT markers. Tonsil epithelial cells isolated from uninfected individuals were treated for 7 days with gp120 and tat; their inactive controls were treated independently. Then cells were examined for expression of E-cadherin, vimentin, N-cadherin, SMAD2, phosphorylated SMAD2, Slug, Snail, Twist1, and ZEB1 by Western blotting. Similar data were obtained in two independent experiments. Immunoblots were performed at least twice, and representative results are shown.

Western blot analysis of EMT markers in tonsil epithelial cells treated with gp120 and tat proteins and their inactive controls showed that gp120 and tat substantially reduced E-cadherin expression and induced vimentin and N-cadherin expression (Fig 5). In contrast, inactive controls of gp120 and tat did not reduce E-cadherin and did not activate vimentin and N-cadherin expression. Furthermore, HIV-1 gp120 and tat activated phosphorylation of SMAD2 and most of the critical transcription factors, including Slug, Snail, Twist1, and ZEB1, which play critical roles in EMT induction (Fig 5).

**Fig 6:**
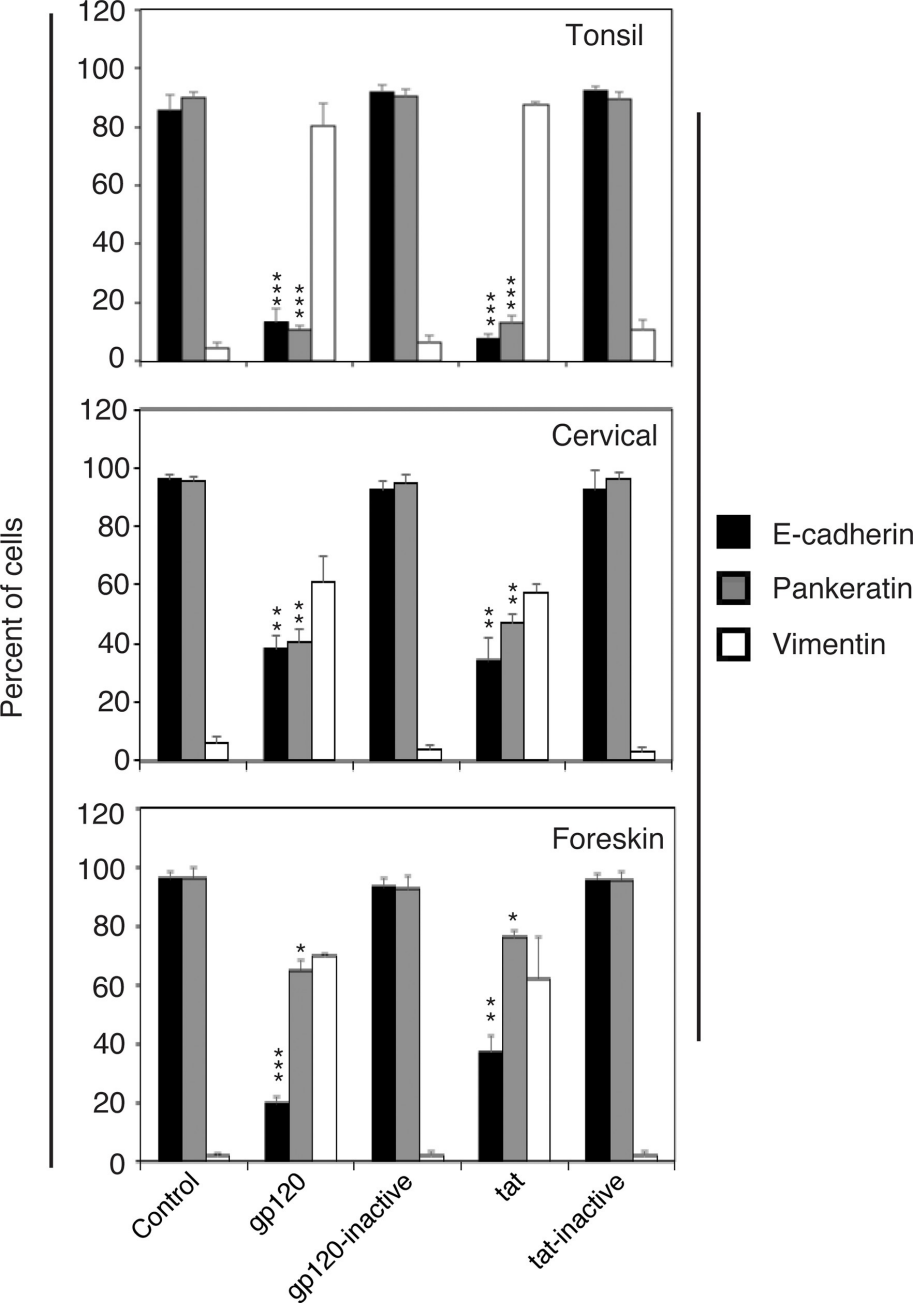
HIV gp120 and tat proteins induce the EMT phenotype in tonsil, cervical, and foreskin epithelial cells. Normal tonsil, cervical, and foreskin keratinocytes were isolated from HIV-negative donors and untreated or treated with HIV tat and gp120; their inactive forms were treated independently. After 7 days, cells were fixed and immunostained for E-cadherin, pancytokeratin, and vimentin. Cells expressing E-cadherin, vimentin, and pancytokeratin were counted in 10 randomly selected regions. Results are presented as a percentage of epithelial cells expressing E-cadherin, vimentin, and pancytokeratin. Data are representative of 2 independent experiments using tonsil, cervical, and foreskin epithelial cells derived from two donors and are shown as the mean ± SD (n = 10). *P<0.05, **P<0.01, ***P<0.001. E-cadherin and pancytokeratin expression were compared with the gp120- and tat-treated and untreated (control) groups.

In the next experiments, we compared HIV-1 tat- and gp120-induced EMT in tonsil, cervical, and foreskin primary epithelial cells by prolonged treatment of cells with HIV proteins tat and gp120. Quantitative analysis of E-cadherin, pancytokeratin, and vimentin expression showed that tat and gp120 proteins inhibited E-cadherin and pancytokeratin and upregulated vimentin expression in all 3 epithelial cell cultures compared to controls (Fig 6). In cervical epithelial cells, reduction of E-cadherin and pancytokeratin by tat and gp120 was ~50%. Interestingly, in foreskin epithelial cells, tat and gp120 led to complete inhibition of E-cadherin expression but did not drastically affect pancytokeratin expression. Nevertheless, induction of vimentin expression was detected in ~70% of foreskin cells. These data indicate that HIV tat- and gp120-induced EMT may occur in oral, cervical, and foreskin epithelial cells.

### HIV cell-free virions via envelope gp120 induce EMT in oral and genital epithelial cells

**Fig 7:**
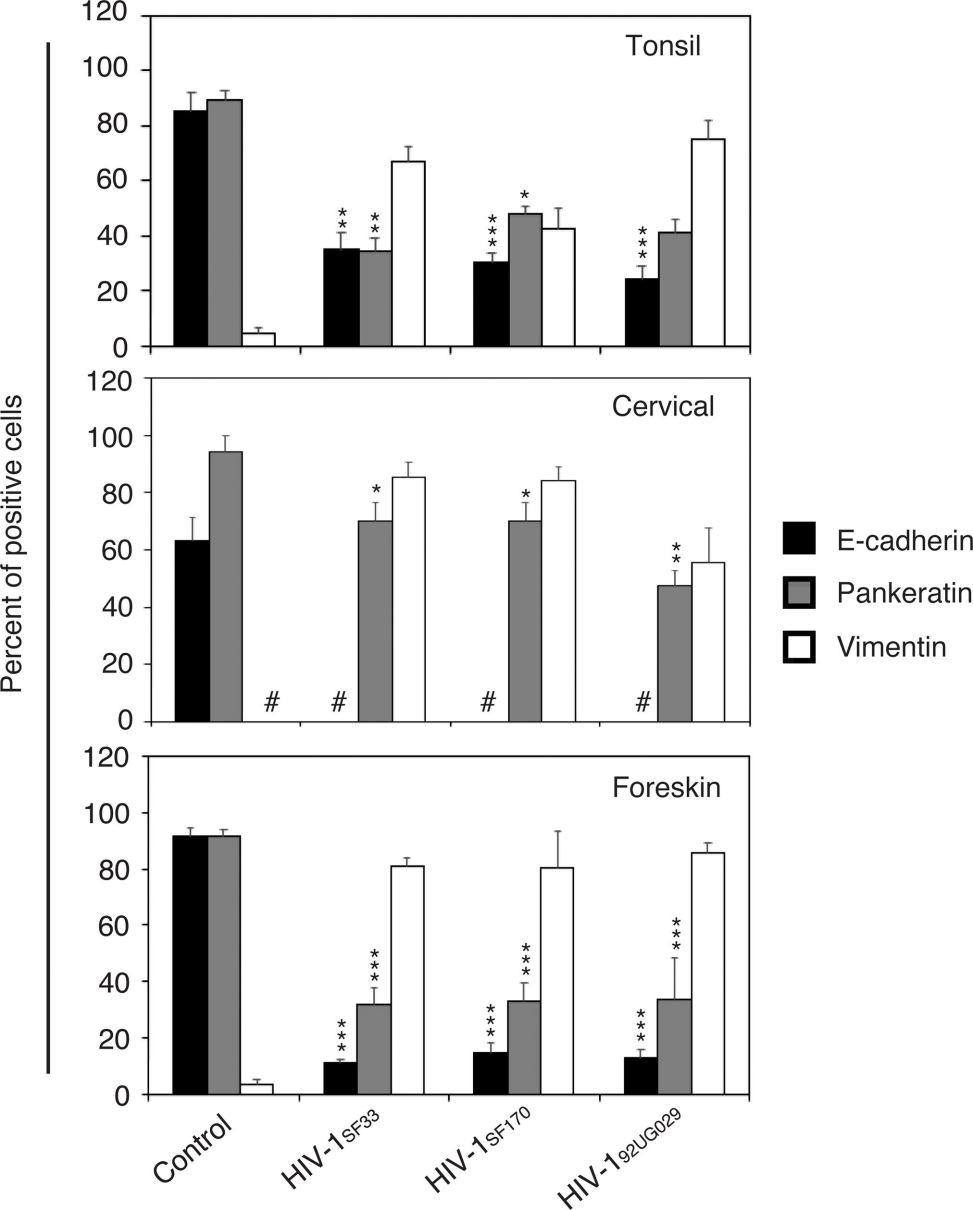
Cell-free HIV virions induce the EMT phenotype in tonsil, cervical, and foreskin epithelial cells. Normal tonsil, cervical, and foreskin keratinocytes isolated from HIV-negative donors were untreated or treated for 7 days with cell-free dual-tropic HIV-1_SF33_, R5-tropic HIV-1_SF170_, and X4-tropic HIV-1_92UG029_ strains. Cells were fixed and immunostained for E-cadherin, pancytokeratin, and vimentin, and cells expressing these proteins were quantitatively evaluated. Results are presented as a percentage of epithelial cells expressing E-cadherin, vimentin, and pancytokeratin. Data are representative of 2 independent experiments using tonsil, cervical, and foreskin epithelial cells derived from two independent donors and are shown as the mean ± SD (n = 10). *P<0.05, **P<0.01, ***P<0.001. E-cadherin and pancytokeratin expression were compared with the gp120- and tat-treated and untreated (control) groups. #, not detected.

The experiments described above showed that HIV protein gp120 induced EMT, suggesting that HIV cell-free virions may also induce EMT. To test this hypothesis, we treated tonsil, cervical, and foreskin keratinocytes with cell-free dual-tropic HIV-1_SF33_, R5-tropic HIV-1_SF170_, and X4-tropic HIV-1_92UG029_ strains for 5 days. All three viruses reduced expression of E-cadherin and pancytokeratin and induced expression of vimentin (Fig 7).

**Fig 8:**
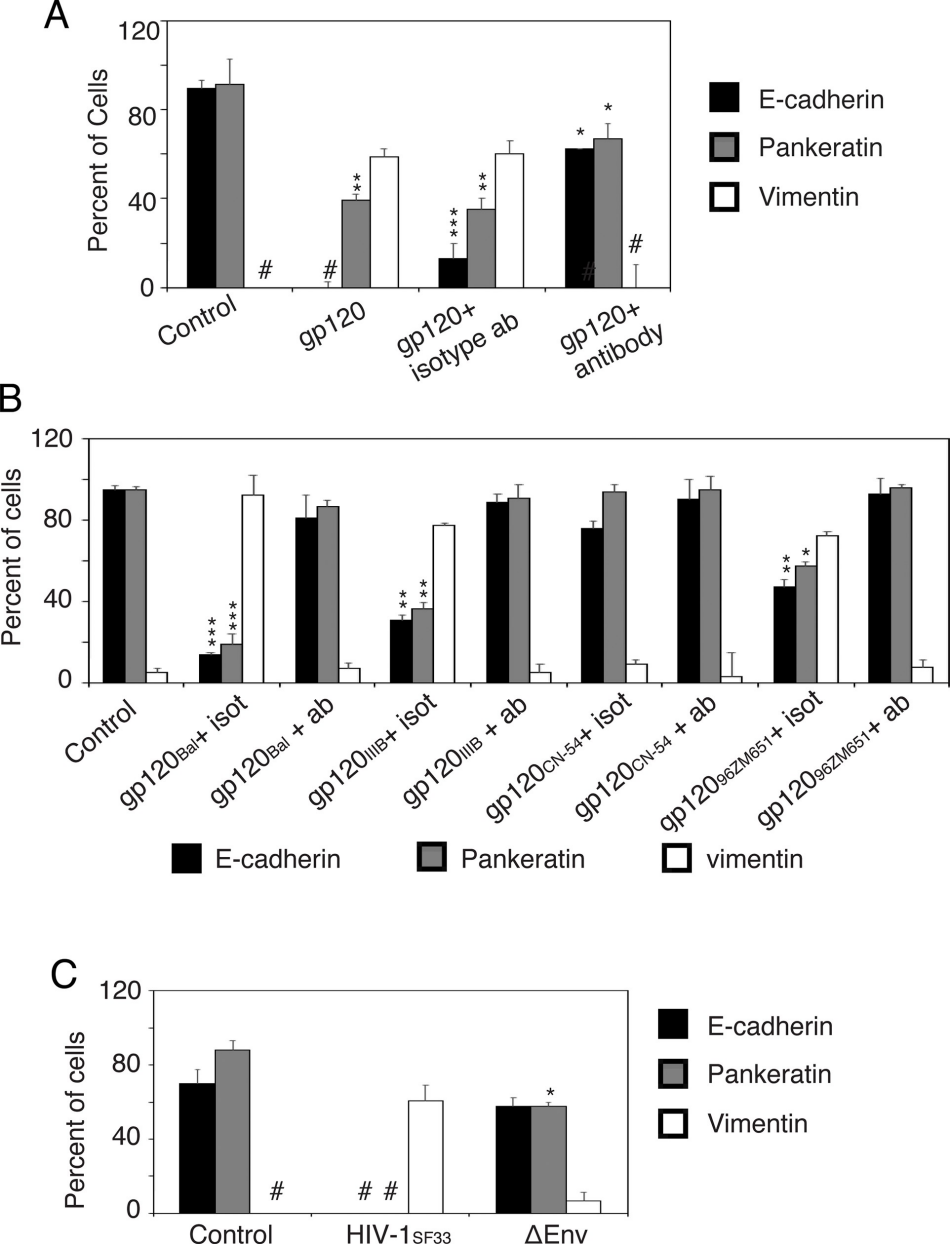
HIV-1 gp120 is critical for EMT induction. (A) gp120 from HIV-1_BAL_ was preincubated with a pool of 4 anti-gp120 antibodies or their isotope controls. Normal tonsil keratinocytes isolated from uninfected donors were untreated or treated with gp120 with neutralizing antibodies or isotype controls. Culture medium was changed every day and after 5 days, cells were quantitatively analyzed for expression of E-cadherin, pancytokeratin, and vimentin. (B) Tonsil keratinocytes were untreated or treated with gp120 from HIV-1_BAL,_ HIV-1_IIIB,_ HIV-1_CN-54_, and HIV-1_JR-CSF_ strains with a pool of neutralizing antibodies or their isotype controls. At day 5, cells were quantitatively analyzed for expression of E-cadherin, pancytokeratin, and vimentin. (C) Tonsil epithelial cells were incubated with HIV-1 Δenv-NL4.3 and HIV-1 NL4.3 viruses, and cells were maintained for 5 days; medium was changed with fresh viruses. After 5 days cells were quantitatively examined for expression of E-cadherin, pancytokeratin, and vimentin. Results are presented as a percentage of E-cadherin-, pancytokeratin-, or vimentin-positive cells. Data are representative of 3 independent experiments using tonsil epithelial cells derived from three donors and are shown as the mean ± SD (n = 10). *P<0.05, **P<0.01, ***P<0.001. E-cadherin and pancytokeratin expression were compared in gp120-treated and untreated control cells. #, not detected.

To further confirm the role of HIV-1 gp120 in induction of EMT, HIV-1_BAL_ gp120 protein were preincubated with a pool of 5 neutralizing antibodies (b12, 2G12, F105, 39F and ID6) or their isotype controls. Then tonsil cells were untreated or treated with gp120, gp120+isotype antibodies, or gp120+ neutralizing antibodies. Cell medium was changed every day, and EMT induction was evaluated after 5 days by quantitation of cells expressing E-cadherin, pancytokeratin, and vimentin. Results revealed that gp120 completely inhibited E-cadherin expression and reduced pancytokeratin expression by ~50% (Fig 8A). Vimentin induction by gp120 was in 50% of cells. In contrast, anti-gp120 antibodies prevented gp120-induced EMT in tonsil epithelial cells; i.e., E-cadherin and pancytokeratin expression were reduced by ~20% and induction of vimentin expression was eliminated. The isotype control antibodies did not affect gp120-mediated induction of EMT in tonsil cells.

To examine if gp120 from different viral strains induce EMT, gp120 proteins from 4 HIV-1 strains—HIV-1_BAL_, HIV-1_IIIB_, HIV-1_CN-54_, and HIV-1_96ZM651_—were preincubated with a pool of 5 neutralizing antibodies or their isotype controls and then added to the tonsil epithelial cells. Quantitation of cells expressing E-cadherin, pancytokeratin, and vimentin showed that gp120 proteins from 3 HIV-1 strains induced EMT, and anti-gp120 antibodies protected cells from gp120-induced EMT (Fig 8B). However, gp120 from HIV-1_CN-54_ did not induce EMT in tonsil epithelial cells.

The role of HIV gp120 in induction of EMT was examined by using HIV-1 Δenv-NL4.3 and HIV-1 NL4.3 viruses. Analysis of EMT of tonsil epithelial cells incubated with these viruses showed that HIV-1 Δenv-NL4.3 failed to induce EMT in contrast to HIV-1 NL4.3, which inhibited E-cadherin and pancytokeratin expression and upregulated vimentin expression (Fig 8C). These findings clearly indicate that envelope protein of gp120 of cell-free HIV-1 induces EMT in tonsil epithelial cells.

**Fig 9:**
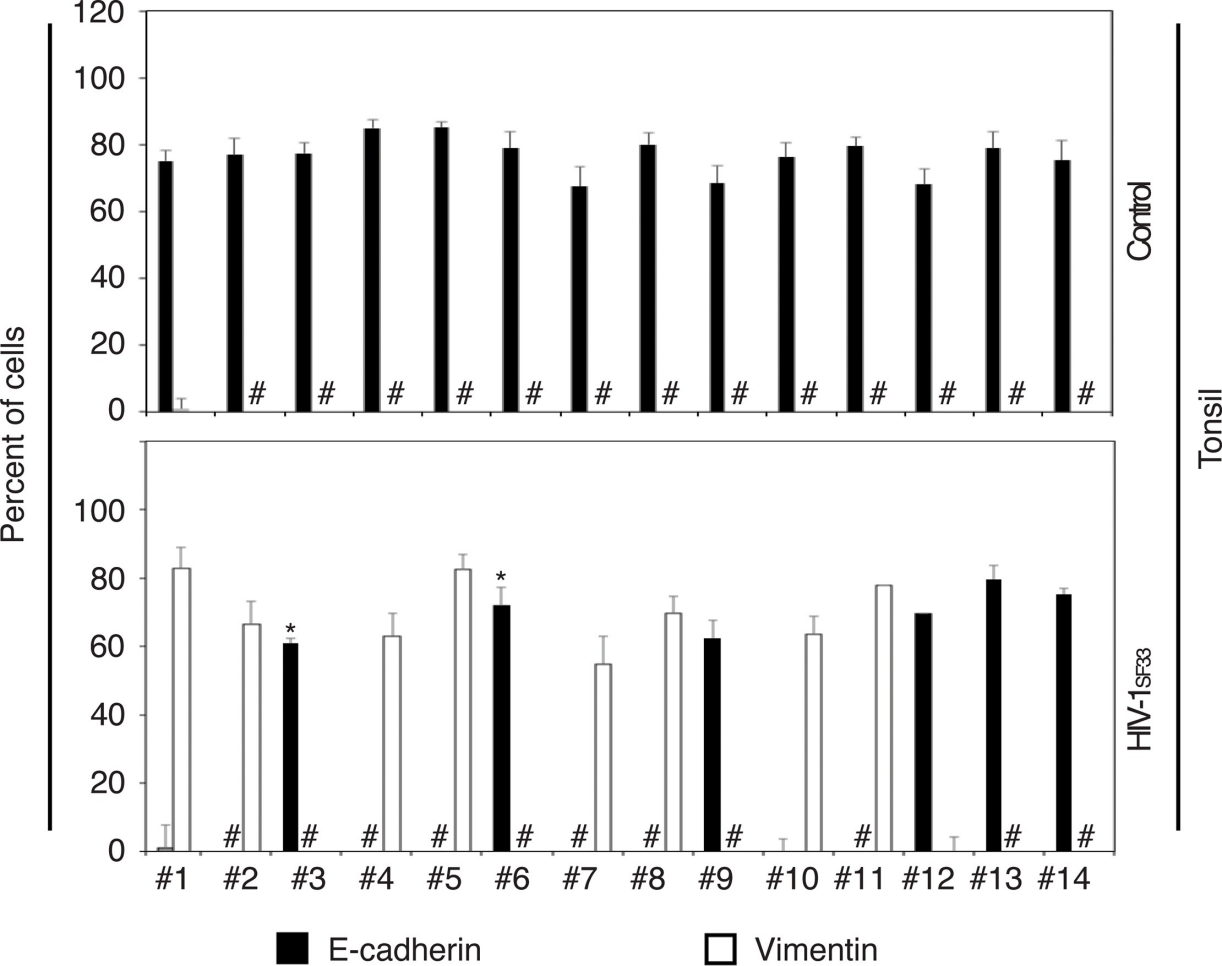
HIV-induced EMT in tonsil epithelial cells isolated from multiple donors. Normal tonsil keratinocytes isolated from 14 uninfected individuals were treated with cell-free HIV-1_SF33_ or untreated for 10 days. Cells were immunostained, and expression of E-cadherin and vimentin were quantitatively evaluated. Data represent the mean ± SD (n = 10). #, not detected. *P<0.05. E-cadherin expression was compared with that of the virus-treated and untreated control cells.

To determine if HIV-induced EMT is a common phenomenon in mucosal oral epithelium, we incubated tonsil epithelial cells from 14 independent donors with HIV_SF33_ cell-free virions for 12 days. Expression of E-cadherin and vimentin showed cell-free HIV-induced downregulation of E-cadherin and upregulation of vimentin in tonsil cells from 8 of 14 donors (57%) (Fig 9), suggesting that HIV-induced EMT may develop in oral mucosal epithelia in 50% of HIV-infected individuals.

### Activation of TGF-β1 and MAPK signaling in oral epithelia of HIV-infected individuals

**Fig 10:**
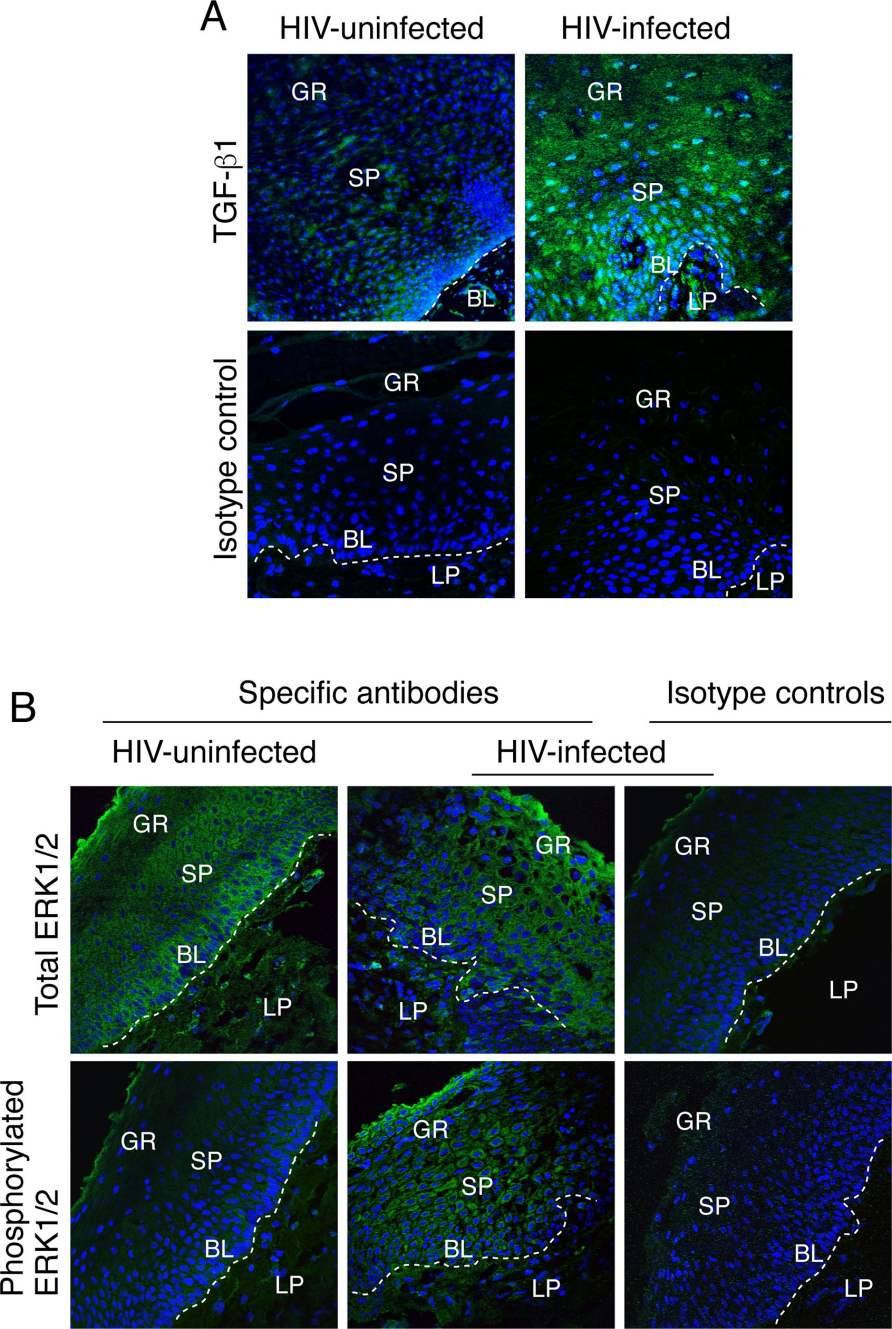
Analysis of MAPK and TGF-β1 signaling in the oral epithelia of HIV-infected and uninfected individuals. Tissue sections from HIV-infected and uninfected individuals were immunostained with antibodies against TGF-β1 (A) or phosphorylated and total ERK1/2 (B) (both in green). Nuclei are stained in blue. EP, epithelium; LP, lamina propria; GR, granulosum; SP, spinosum; BL, basal. Representative images of three independent biological replicates are shown.

To examine the status of TGF-β1 and MAPK signaling in oral epithelia of HIV-infected and uninfected individuals, we immunostained tissue sections of buccal biopsy samples from 3 HIV-infected and 3 uninfected individuals for TGF-β1 and for phosphorylated and total ERK1//2. Immunofluorescence analysis showed substantially higher levels of TGF-β1 expression in buccal epithelium of HIV-infected individuals than that in uninfected individuals (Fig 10A). Furthermore, ERK1/2 was highly phosphorylated in the oral epithelia of HIV-infected individuals but not in that of uninfected individuals (Fig 10B). Our recent work showed that HIV-tat/gp120-induced activation of MAPK in tonsil epithelial cells leads to reduction of E-cadherin expression, suggesting the role of HIV-associated MAPK activation in EMT [21, 22]. A similar trend was observed in all 3 biopsy tissues of HIV-infected individuals. These data indicate that TGF-β1 and MAPK signaling in the oral epithelia of HIV-infected individuals are activated, consistent with their potential roles in the induction of EMT.

**Fig 11:**
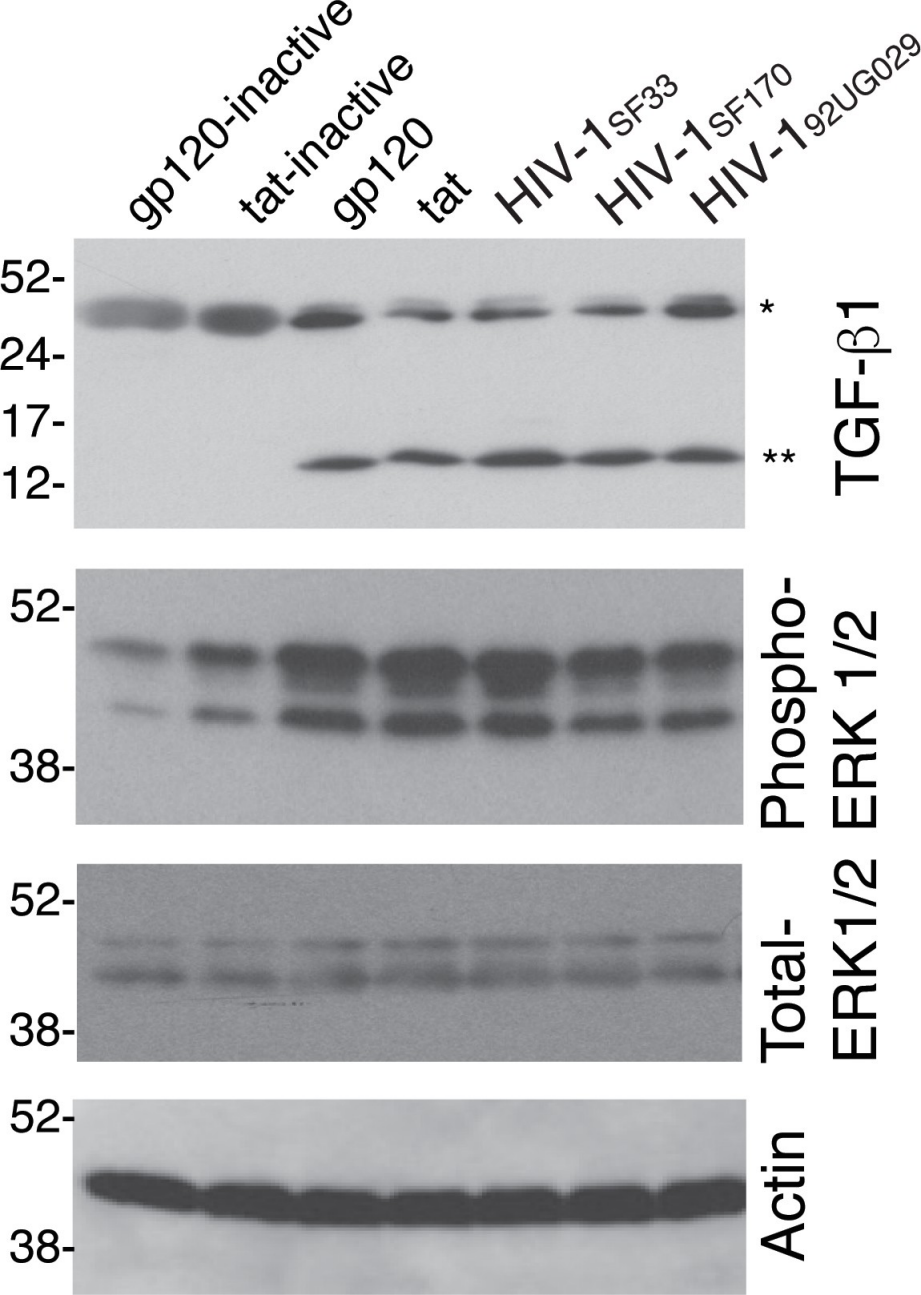
Activation of TGF-β1 and MAPK signaling by HIV-1 tat, gp120, and cell-free virions in tonsil cells with the EMT phenotype. Normal tonsil keratinocytes isolated from uninfected individuals were treated for 7 days with gp120, tat, and their inactive forms, and cell-free HIV-1_SF33_, HIV-1_SF170_, and HIV-1_92UG029_ strains. Cells were lysed and used to detect TGF-β1, phosphorylated and total ERK1/2, and actin by Western blotting using specific antibodies. *Precursor; **mature active form. A representative Western blot is shown from two independent experiments.

HIV-induced activation of TGF-β1 and ERK1/2 signaling was also examined in tonsil keratinocytes in the in vitro experiments. Tonsil keratinocytes were treated with gp120 and tat proteins and their inactive forms, as well as with cell-free virions of dual-tropic HIV-1_SF33_, R5-tropic HIV-1_SF170_, and X4-tropic HIV-1_92UG029_ strains for 10 days. After confirmation of EMT induction by light microscopy, cells were examined for TGF-β1 and ERK1/2 activation by Western blot assay. Data showed that tonsil keratinocytes incubated with active gp120 and tat as well as cell-free virions of HIV-1_SF33_, HIV-1_SF170_, and HIV-1_92UG029_ strains induce activation of TGF-β1 by the resulting formation of cleaved mature TGF-β1 protein (Fig 11). In contrast, cells treated with inactive gp120 and tat proteins showed only the inactive form of TGF-β1. Phosphorylated ERK1/2 was also detected, mostly in cells treated with gp120, tat, and cell-free virions. These data clearly demonstrated that HIV proteins gp120 and tat are responsible for the activation of TGF-β1 and MAPK signaling, which are critical for induction of the EMT phenotype.

**Fig 12:**
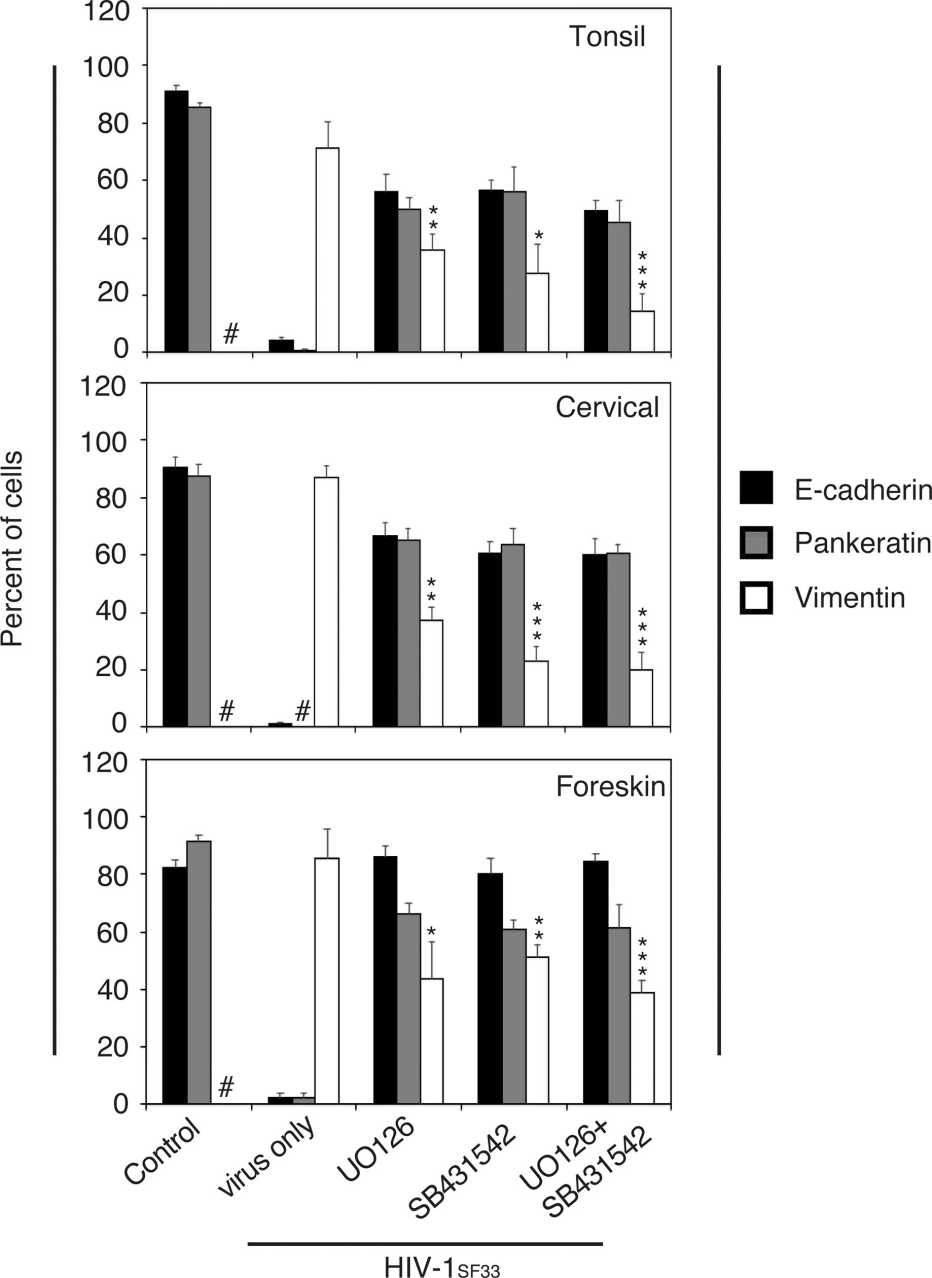
Inhibition of TGF-β1 and MAPK signaling reduces HIV-induced EMT phenotype in tonsil, cervical, and foreskin epithelial cells. Normal tonsil, cervical, and foreskin keratinocytes isolated from uninfected individuals were incubated for 5 days with cell-free HIV-1_SF33_ in the presence or absence of MAPK UO126 or TGF-β1 SB431542 inhibitors. Cells were immunostained for E-cadherin, pancytokeratin, and vimentin, and their expression was quantitatively analyzed. Results are presented as a percentage of E-cadherin-, pancytokeratin-, or vimentin-positive cells. Data are representative of 2 independent experiments using tonsil, cervical, and foreskin epithelial cells derived from two donors and are shown as the mean ± SD (n = 10). *P<0.05, **P<0.01, ***P<0.001. Vimentin expression were compared in cells incubated with HIV or HIV plus drugs. #, not detected.

To confirm the functional roles of TGF-β1 and ERK1/2 signaling in HIV-induced EMT, we treated tonsil, cervical, and foreskin keratinocytes with nontoxic concentrations of inhibitors of MAPK UO126, TGF-β1 SB431542, and their combination. Drug-treated and untreated cells were incubated with cell-free HIV-1_SF33_ virions. At 5 days after treatment, cells were immunostained for E-cadherin, pancytokeratin, and vimentin. Quantitative analysis showed that cells incubated with only HIV virions led to complete inhibition of E-cadherin and pancytokeratin expression and significant induction of vimentin expression; i.e., ~75–80% of cells expressed vimentin (Fig 12). In contrast, cells incubated with HIV virions and inhibitors of TGF-β1 or/and MAPK show reduction of E-cadherin and pancytokeratin expression in ~20–40% of cells. Moreover, reduction of vimentin expression was highly significant, i.e., 50–80%. Thus, inhibition of TGF-β1 and ERK1/2 signaling significantly prevented EMT induction in tonsil, cervical, and foreskin epithelial cells by cell-free virions, indicating the critical roles of TGF-β1 and ERK1/2 signaling in HIV-induced EMT.

### Transmigration of HIV-induced EMT cells

**Fig 13:**
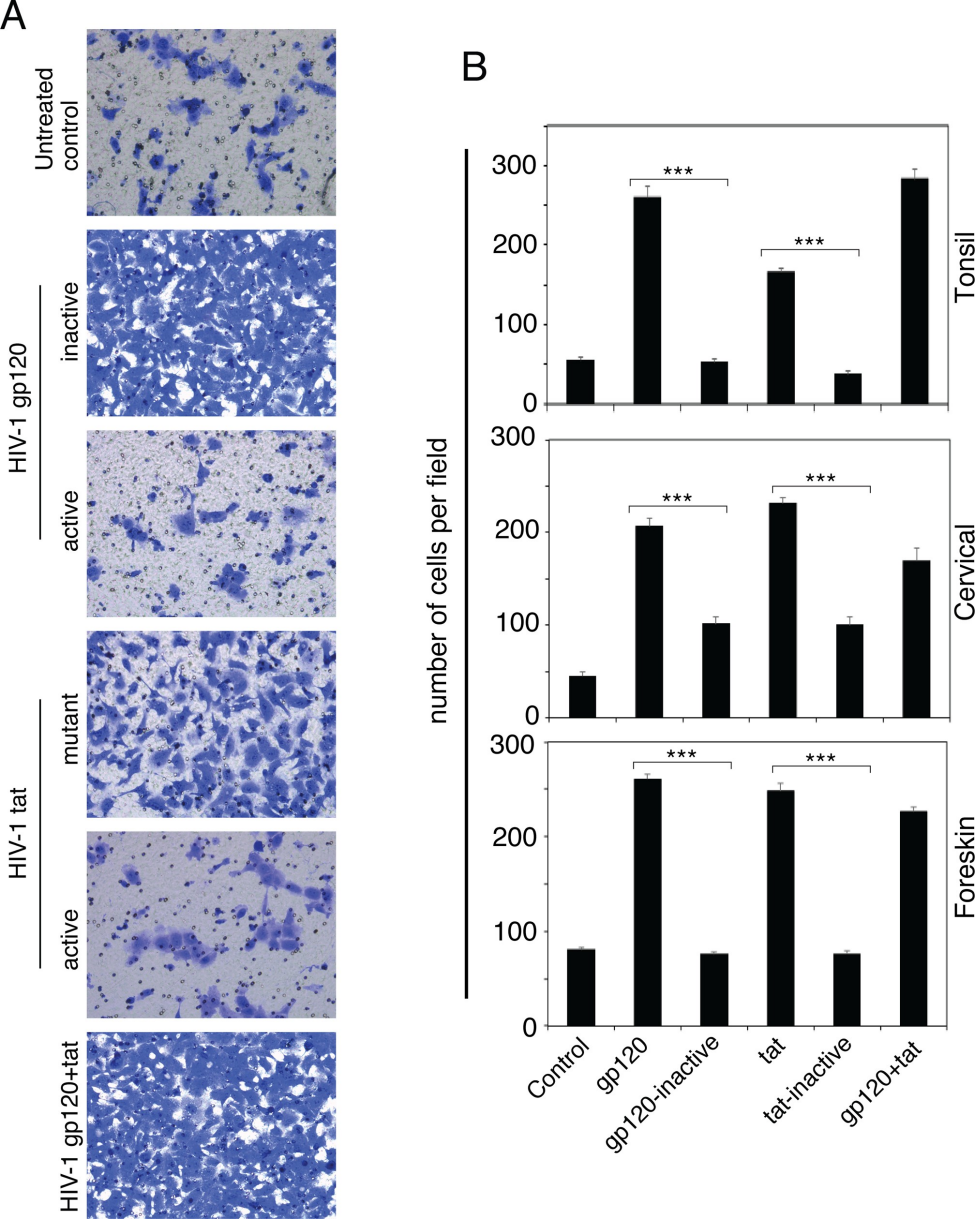
Transmigration and invasion of HIV gp120- and tat-induced EMT cells. Normal tonsil, cervical, and foreskin keratinocytes were isolated from uninfected donors and treated for 5 days with gp120 and/or tat and their inactive forms. Untreated cells served as a control. Transmigration of cells was examined in collagen-coated Transwell inserts. Cells were stained (A) and quantitatively evaluated (B). Transmigrated invasive cells were counted in 10 randomly selected regions. Results are presented as average number of cells per field. Data are representative of 2 independent experiments using tonsil, cervical, and foreskin epithelial cells derived from two donors and are shown as the mean ± SD (n = 10). ***P<0.001. Cell numbers were compared with those of the gp120- and tat-treated cells and their inactive forms (control).

It is well known that EMT cells are highly motile and have intensive migratory activity [36–39]. To determine if tonsil, cervical, and foreskin cells incubated with HIV have migratory activity, we incubated cells with gp120 and tat proteins and their inactive forms for 5 days. Then, cells were seeded in Transwell inserts coated with collagen for 2 days. KGM medium was added to the lower chamber with 10% serum as a chemoattractant. Transmigration of cells via collagen-coated membrane pores was visualized by Giemsa staining (Fig 13A), and the rate of transmigration and invasion was measured after 24 h by counting cells in the lower side of the membranes. Quantitative analysis showed that gp120 and tat proteins increased transmigration of cells by four- to fivefold compared to untreated cells or cells treated with inactive gp120 or tat (Fig 13B). These data indicate that HIV gp120- and tat-induced EMT cells transmigrated via collagen-coated membranes; i.e., they are highly motile and invasive, which are typical features of the EMT phenotype.

## Discussion

Our findings clearly demonstrate that prolonged interaction of HIV proteins gp120 and tat and cell-free HIV virions with tonsil, cervical, and foreskin epithelial cells leads to development of the EMT phenotype in these cells. Loss of E-cadherin and upregulation of vimentin expression in the buccal epithelial tissues and their in vitro isolated keratinocytes from HIV-infected individuals suggest that HIV infection may play a critical role in EMT induction in oral epithelium in vivo. This idea is fully supported by the induction of EMT by HIV gp120 and tat and cell-free virions in vitro in the tonsil, cervical, and foreskin keratinocytes isolated from HIV-negative individuals.

A substantial reduction of E-cadherin and pancytokeratin expression and induction of vimentin in oral biopsy samples from ART-untreated individuals with higher viral load suggest that HIV infection may play a direct role in EMT induction. However, the moderate level of EMT induction also was observed in oral mucosal epithelium of ART-treated individuals, suggesting that oral intramucosal Langerhans cells, macrophages, and CD4+ T cells in the ART-treated patients may still have replicating virus, as shown in our previous work [7]. It is possible that ART may not completely eliminate HIV from intramucosal immune cells [7] due to a lower level of penetration of drugs into solid tissues [93–96]. We also cannot completely rule out the possible contribution of HIV-associated systemic elevation of proinflammatory cytokines in EMT induction [97–100].

**Fig 14:**
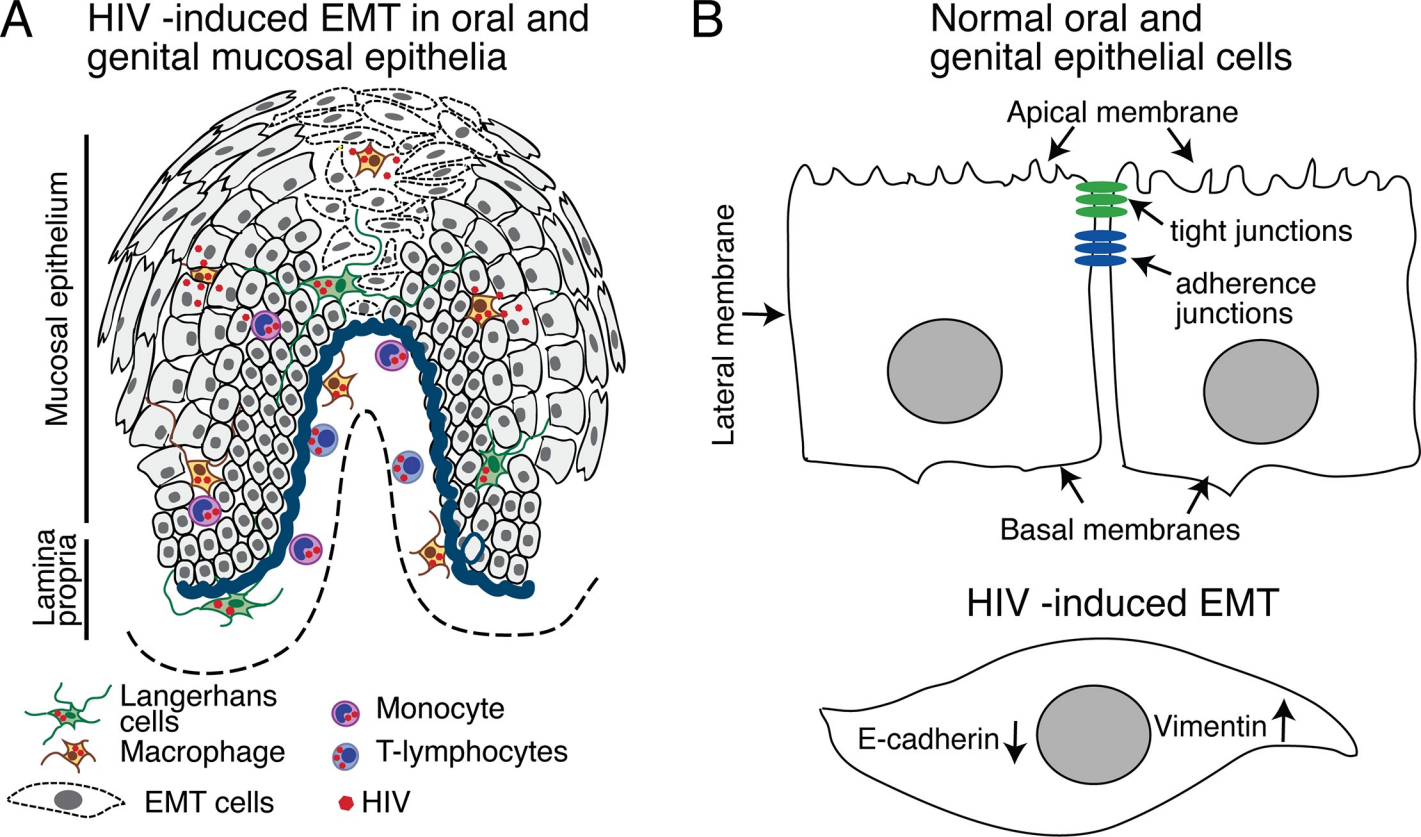
Model of HIV-induced EMT. In HIV-infected individuals, HIV-infected CD4 lymphocytes, monocyte/macrophages, and Langerhans cells migrate into oropharyngeal, cervical, and foreskin epithelia and produce cell-free virions and secrete HIV proteins tat and gp120 in the mucosal environment (A). Interaction of cell-free virions and viral proteins with epithelial cells activates MAPK and TGF-β, leading to induction of the EMT phenotype: Epithelial cells lose their cobblestone-like morphology and acquire a spindle-like shape (B). Epithelial markers, including E-cadherin expression, are lost and the mesenchymal marker vimentin expression is induced. EMT cells lose adherens and tight junctions and cell polarity, leading to impairment of the epithelial barrier. The lack of epithelial junctions causes the opening of the paracellular space for penetration of viral, bacterial, fungal, and other pathogens. HIV-induced EMT cells of mucosal epithelia are highly motile and may migrate through basement membranes. If HIV-induced EMT occurs in the premalignant or malignant mucosal epithelia, it may accelerate the neoplastic process, leading to more migration and invasion of cancer cells.

If HIV infection is indeed a biologically relevant contributor to EMT, then HIV proteins should be present in the mucosal environment. Indeed, cell-free HIV-1 virions and viral DNA/RNA can be isolated from oral and genital mucosal epithelium, as well as the saliva and cervicovaginal secretions of HIV-positive individuals [7, 101–114]. HIV-infected lymphocytes, macrophages, and Langerhans cells were detected in the oral and genital mucosal epithelia of HIV-infected individuals, including those receiving ART [7, 101, 102, 107, 108, 112–115]. Secretion of HIV tat into blood has been shown [86, 87, 89]. HIV-1 gp120 and tat have also been detected in blood, saliva and lymphoid tissues [7, 88, 116–120]. Mucosal epithelium may therefore be exposed to cell-free HIV and tat and gp120 from multiple sources, including saliva, cervicovaginal secretions, and circulating immune cells (Fig 14). Mucosal epithelium may also serve as an HIV reservoir [113], as well as a source of proteins that induce EMT in the setting of HIV infection.

HIV-1 gp120- and tat-induced EMT cells express most critical transcription factors, including Slug, Snail, Twist1 and ZEB1, which downregulate epithelial markers and upregulate mesenchymal markers. Expression of these transcription factors was correlated with expression of the mature form of TGF-β1; i.e., gp120- and tat-induced EMT is due to activation of TGF-β1 signaling. Blood levels of TGF-β are elevated in HIV-positive individuals [121–124]. HIV-1 gp120 and tat induce TGF-β expression in macrophages, CD4 and CD8 lymphocytes, and natural killer cells [89, 125–127]. HIV-associated elevation of TGF-β expression within the mucosal environment may play a critical role in induction of EMT in mucosal epithelia. We have shown that HIV-1 gp120 and tat activate expression of matrix metallopeptidase 9 through MAPK and NF-κB signaling [22]. It is possible that HIV-1 gp120- and tat-activated matrix metallopeptidase 9 may contribute to cleavage of the TGF-β precursor protein, generating formation of its C-terminal mature fragment, which binds to receptors and activates TGF-β signaling [128, 129].

Both gp120 and tat activate MAPK signaling, which also plays a critical role in EMT induction. TGF-β expression is activated by the AP-1 transcription factor [89, 130], which is induced by MAPK signaling [89, 131, 132], and this may lead to elevation of TGF-β expression. HIV-1 gp120 binds to the chemokine receptors CXCR4 and CCR5 and *galactosylceramide* (GalCer), inducing MAPK activation [133–135] [24, 133, 134, 136]. HIV gp120 also binds to heparan sulfate proteoglycans (HSPG) [8, 9, 137–140], which bind TGF-β superfamily proteins leading to activation of TGF-β signaling [141]. Reduction of gp120-mediated EMT by anti-gp120 antibodies suggested that gp120 interaction with one or more receptors on the epithelial surface could be critical for the induction of EMT.

HIV tat binds to α5β1, α5β3, and αvβ3 integrins [142–146] and induces ras-dependent activation of MAPK [91]. We and others have shown that tonsil and genital epithelia express receptors CXCR4 and CCR5, GalCer, and β1 and αv integrins [8, 9, 137–140, 147].

The lack of difference between X4, R5, and dual-tropic HIV-1 viruses in EMT induction in tonsil, cervical, and foreskin cells suggests that gp120 interaction with these epithelial cells is not dependent on viral tropism. However, the lack of gp120-induced EMT induction by one of 4 HIV-1 strains suggests that some HIV-1 strains may have altered gp120, which may not bind its epithelial receptors and induce EMT.

MAPK signaling may also play a direct role in EMT induction by phosphorylation of Smad2/3 and TWIST1 [56–63].

HIV-1 tat may penetrate into cells and tissues through its protein transduction domain, based on the amino acids arginine and lysine, which facilitate protein internalization into cells and tissues by multiple mechanisms, including endocytosis and macropinocytosis [148–152]. Indeed, we have shown that recombinant HIV-1 tat is internalized into stratified oral epithelium [7]. Intracellular tat may increase TGF-β expression by interacting with AP-1 [153], suggesting that tat may activate TGF-β signaling by multiple mechanisms.

HIV proteins may also induce EMT through TGF-β- and MAPK- independent mechanisms. HIV tat binding to integrins facilitates activation of epidermal growth factor receptor (EGFR) [91]. HIV gp120 binding to GalCer of epithelial cells induces intracellular calcium elevation, which leads to the activation of protein kinase C [24, 154, 155] and subsequent activation of EGFR [156, 157]. Both tat- and gp120-induced activation of EGFR upregulate expression of the transcription factor STAT3, which subsequently activates Twist1 and downregulates E-cadherin expression, initiating EMT.

HIV-1 gp120 and tat activate expression of proinflammatory cytokines, including tumor necrosis factor alpha [158, 159], which may stimulate EMT [97–100].

HIV-1 gp120- and tat-induced EMT in tonsil, cervical, and foreskin epithelial cells indicates that HIV-induced EMT could be common in oral and genital epithelia. Induction of EMT by HIV-1 recombinant gp120 and cell-free HIV virions revealed that the initial interaction of the cell-free virions envelope with mucosal epithelium from uninfected individuals may lead to the loss of epithelial junctions and paracellular penetration by HIV. However, the lack of HIV-1_SF33_ cell-free virion-induced EMT in 45% of tonsil keratinocytes isolated from independent donors suggests that not all mucosal epithelial cells have critical epithelial receptors or signaling molecules for gp120.

HIV-1 gp120- and tat-induced EMT during HIV/AIDS disease may reduce the barrier functions of oral and genital mucosal epithelium, leading to the spread of viral, bacterial, fungal, and other pathogens. In the intact epithelium the assembled epithelial junction may sequester receptors for viruses, reducing their infection and mucosal transmission. HIV-induced EMT may liberate hidden receptors owing to the disassembly of epithelial junctions. Indeed, we reported that nectin-1, a receptor for herpes simplex virus-1, is hidden within the adherens and tight junctions of oral epithelium, and HIV-induced disruption of epithelial junctions exposes nectin-1 to herpes simplex virus-1, facilitating rapid viral infection and spread [21].

HIV-induced disruption of epithelial junctions via EMT facilitates paracellular penetration of oncogenic human papillomavirus (HPV) [7]. Moreover, HIV gp120- and tat-induced transmigration of EMT cells suggests that, if this occurs within the HPV-infected environment, it may significantly accelerate invasion of HPV-infected malignant cells, leading to the progression of HPV neoplasia. Indeed, our ongoing studies show that HIV gp120 and tat substantially increase migration and invasion of HPV-infected cervical cancer cells through EMT mechanisms (manuscript in preparation).

HIV has been shown to cause EMT in renal epithelium. HIV infection is associated with kidney failure due to severe nephropathy, characterized by the loss of the renal epithelial phenotype and acquisition of mesenchymal features, including dedifferentiation, depolarization, and proliferation [160–167]. Accumulating evidence indicates that HIV-associated nephropathy is caused by EMT [166] and that HIV infection may play a critical role in the induction of EMT [160–165, 168, 169]. HIV replication has been observed in renal epithelial cells [169, 170]. Studies using HIV transgenic mice have shown that renal epithelial cells express HIV mRNA and develop the EMT phenotype with decreasing E-cadherin expression [160, 171, 172]. Transgenic mice expressing the HIV proteins nef or tat show EMT-like changes in the renal epithelium [173]. An HIV nef-induced EMT-like phenotype has been seen in experiments with renal epithelial cells in vitro [173–176].

HIV-associated reduction of E-cadherin expression have been shown in lung and gut epithelia [177, 178] suggesting HIV-induced EMT may also take place in other epithelial organs in HIV/AIDS disease, such as skin, nasopharyngeal mucosal epithelium, and liver. HIV-induced EMT may accelerate the epithelial neoplasia associated with other oncogenic viruses, such as Epstein Barr virus (EBV), Kaposi sarcoma-associated herpesvirus, and hepatitis C (HCV) and B (HBV) viruses. It is well known that HIV coinfection is common with EBV, Kaposi sarcoma-associated herpesvirus, HCV, and HBV with the development of mutual pathogenesis for these copathogens [179–181].

In summary (Fig 14), we have shown that HIV-1 gp120 and tat proteins induce EMT in oral and genital mucosal epithelia via activation of TGF-β and MAPK signaling. EMT-associated changes to the integrity of mucosal epithelia may reduce a wide spectrum of physiologic functions of mucosa, including barrier, transport, secretion, and maintenance of the innate and adaptive immune response by recruiting monocyte/macrophages, Langerhans cells, and T and B lymphocytes [6, 182, 183]. Importantly, HIV-induced EMT may occur within premalignant cells, which may accelerate the progression of neoplastic processes. Inhibition of TGF-β and MAPK signaling pathways may inhibit development of HIV-induced EMT of oral and genital mucosal epithelia, preserving their normal barrier and other functions.

## Materials and methods

### Ethics statement

This study was conducted according to the principles expressed in the Declaration of Helsinki and was approved by the Committee on Human Research of the University of California–San Francisco (IRB approval #s 10–03277 and 19–27275). All human subjects gave informed written consent for the collection of tissue samples. The parents provided informed consent for all minors.

### Collection of buccal tissues

Buccal biopsy samples from HIV-uninfected and HIV-infected individuals were collected by the UCSF Oral AIDS Center. The main criteria of tissue collection were the lack of clinically detectable HPV-, human cytomegalovirus (HCMV)-, HSV- and EBV-specific lesions and inflammation.

### Viruses, viral proteins, and cells

Laboratory-adapted dual-tropic (X4-R5) HIV-1_SF33_ and the primary isolates R5-tropic HIV-1_SF170_ and X4-tropic HIV-1_92UG029_ were grown in peripheral blood mononuclear cells, which were isolated from heparinized blood using a Ficoll-Paque Plus density gradient (Sigma). Cells were activated with 2.5 μg/ml phytohemagglutinin (Sigma) and 1 μg/ml interleukin-2 (BD Biosciences) for 3 days. Viral stocks were purified using the Amicon Ultra-15 ultracentrifugation filtration system (Millipore). Viral stocks were titered by p24 concentration using HIV-1 p24 ELISA (PerkinElmer) according to the manufacturer’s instructions.

Recombinant HIV-1_Bal_ tat and its inactive form were purchased from ImmunoDX, LLC (Woburn, MA). Inactive tat was created through substitution of its basic arginine-rich domain at the 49–57 aa and the integrin-binding RGD motif in the C terminus with alanines [90–92]. Recombinant gp120 proteins from HIV-1_BAL_, HIV-1_IIIB_, HIV-1_CN-54_, and HIV-1_96ZM651_ strains were provided by the NIH AIDS Reagent Program. gp120 was inactivated by incubation at 85°C for 30 min [184–186]. All HIV proteins were stored at –80°C in the dark before use.

Wild-type and HIV-1 NL4.3 and HIV-1 NL4.3-E (HIV-1 Δenv) lacking envelope protein were obtained from the NIH AIDS Reagent Program. HIV-1 NL4.3 and HIV-1 NL4.3-E Δenv were propagated in HEK293 cells, purified using the Amicon Ultra-15 ultracentrifugation filtration system (Millipore), and titered by p24 concentration using HIV-1 p24 ELISA.

Primary tonsil epithelial keratinocytes were established from tonsil tissue from 20 HIV-negative children <5 years of age after routine tonsillectomy. Primary cervical keratinocytes were established from ectocervical tissue specimens from 4 HIV-negative donors. Foreskin keratinocytes from three independent donors were obtained from Lonza (Hayward, CA). Keratinocytes were grown in keratinocyte growth medium (KGM gold) (Lonza). Epithelial cell purity and absence of mesenchymal cells were determined with a cocktail of antikeratin antibodies containing Ab-1 and Ab-2 (Thermo Fisher Scientific). Only epithelial cell populations with 100% keratin were used. Keratinocytes were used at early passages and frozen in liquid nitrogen.

### Treatment of tonsil, foreskin, and cervical epithelial cells with cell-free HIV virions and HIV proteins gp120 and tat

Cervical, foreskin, and tonsil epithelial cells were treated with active tat and gp120, inactive mutant tat, and heat-inactivated gp120 at a concentration of 10 ng/ml (each) for 5–12 days. Cells were exposed to dual-tropic HIV-1_SF33_, R5-tropic HIV-1_SF170_, and X4-tropic HIV-1_92UG029_ at a concentration of 10 ng/ml of p24. Culture medium was changed daily to add fresh virus or proteins. One set of cells was treated with MAPK inhibitor U0126 (Sigma) or with TGF-β1 inhibitor SB431542 (Tocris Bioscience) at 10 μM each. The absence of a toxic effect by virus, tat, gp120, U0126, or SB431542 was confirmed by the MTT cell viability assay (Biotium). Cervical, foreskin, and tonsil epithelial cells were also exposed to HIV-1 NL4.3 and NL4.3-E Δenv viruses at 10 ng/ml.

To neutralize the EMT induction effect of gp120 in tonsil epithelial cells, gp120 from HIV-1_BAL_, HIV-1_IIIB_, HIV-1_CN-54_, and HIV-1_96ZM651_ were incubated with a pool of 5 neutralizing antibodies—b12, 2G12, F105, 39F and ID6—or pool of their isotype controls for 1 h at 37°C at 1 μg/ml of each. Then gp120 with antibodies were added to the tonsil epithelial cells. Cell culture medium was changed every day with fresh proteins and antibodies, and after 5 days, cells were quantitatively analyzed for expression of E-cadherin, pancytokeratin, and vimentin. gp120 and antibodies were obtained from NIH AIDS Reagent Program.

### Immunofluorescence assay

For immunofluorescence assays, cells or tissue sections were fixed with 4% paraformaldehyde and 2% sucrose in PBS for 5 min, and then permeabilized with 0.01% Triton X-100 in 4% paraformaldehyde for 5 min. Normal donkey serum (5%) in PBS was used to prevent nonspecific binding. E-cadherin was detected using either rabbit or goat antibodies (Vector Laboratories and R&D Systems, respectively). Vimentin was detected using goat antibodies (Millipore), and pankeratin was detected using rabbit antibodies (Life Technologies). Primary isotype control antibodies were used as a negative control to confirm the specificity of each antibody. Primary antibodies were incubated for 1.5 h. Secondary antibodies used in this assay include Dylight 488, Dylight 594 (Vector Laboratories), and Alexa Fluor 594 (Jackson ImmunoResearch Laboratories). Cell nuclei were counterstained with DAPI (Molecular Probes). Images were captured with a Nikon Eclipse E400 fluorescence microscope (Nikon) at magnification 200x. Quantitative analysis of EMT was undertaken by counting E-cadherin-, vimentin-, and pancytokeratin-expressing cells relative to the total number of cells per image in 10 separate, randomly chosen fields on each slide (n = 10). Cell counting was performed independently by 2 investigators (KL, WM).

### Western blot assay

Cells were extracted with 1.0% Triton X-100 buffer (150 mM NaCl, 10 mM Tris/HCl, pH 8.0, and a cocktail of protease inhibitors). Proteins were separated on an SDS-polyacrylamide gel with a 4–20% gradient. The following antibodies were used: rabbit antibody to E-cadherin (R&D); rabbit antibodies against N-cadherin, vimentin, SMAD2, Slug, Snail, and ZEB1 (Cell Signaling); rabbit antibodies against phosphorylated SMAD2 (Abcam); mouse monoclonal antibodies against TGF-β1 (Thermo Fisher Scientific and Abcam); rabbit antibodies against ERK1/2 total and ERK1/2 phosphorylated. An equal protein load was confirmed by the use of beta-actin (Ambio).

### Transmigration assay

In vitro transmigration and invasion assays were performed using the Collagen Cell Invasion Assay-Colorimetric (8 μM) (EMD Millipore) according to the manufacturer’s protocol. Cells were treated with HIV-1 gp120 and tat proteins or their inactive controls for 5–7 days, and 5x10^4^ cells/insert were seeded in the collagen-coated inserts in basal KBM medium without supplements. KMB alone or KGM containing 10% fetal bovine serum as chemoattractant was added to the lower chamber. Cell migration and invasion were evaluated 24 h later using light microscopy to count individual cells that invaded the collagen inserts. To quantify cell migration/invasion, cell numbers on 10 randomly selected fields were counted under various experimental conditions; data are presented as average number of cells per field.

### Statistical analysis

Statistical comparisons were made by a two-tailed Student’s t-test. A p value of <0.05 was considered significant. Results are expressed as mean ± SD.

## Supporting information

S1 Table Antiretroviral therapy status, viral load and CD4 counts of oral biopsy of donors. (DOCX)
