# Supplementary material for: HIV-1 proteins gp120 and tat induce the epithelial–mesenchymal transition in oral and genital mucosal epithelial cells
Source: PLoS One. 2019 Dec 23;14(12):e0226343. doi: 10.1371/journal.pone.0226343 (PMC6927651; doi:10.1371/journal.pone.0226343)
Supplement: S1 Table — (DOCX) [file pone.0226343.s001.docx]

| Tissues | Antiretroviral therapy | HIV viral load (copies/ml) | CD4 cells/ml |
| --- | --- | --- | --- |
| #1 | Yes | Not detected | 300 |
| #2 | Yes | 76 | 29 |
| #3 | Yes | Not detected | 715 |
| #4 | Yes | 250 | 209 |
| #5 | No | 115,000 | 333 |
| #6 | No | 41,000 | 346 |
| #7 | No | 22,134 | 305 |
| #8 | No | 23,050 | 409 |
| #9 | No | 200,000 | 75 |
| #10 | No | 260,000 | 34 |
|  |  |  |  |
